# *Sphagnum capillifolium* holobiont from a subarctic palsa bog aggravates the potential of nitrous oxide emissions

**DOI:** 10.3389/fpls.2022.974251

**Published:** 2022-09-07

**Authors:** Yanxia Nie, Sharon Yu Ling Lau, Xiangping Tan, Xiankai Lu, Suping Liu, Teemu Tahvanainen, Reika Isoda, Qing Ye, Yasuyuki Hashidoko

**Affiliations:** ^1^Key Laboratory of Vegetation Restoration and Management of Degraded Ecosystems, South China Botanical Garden, Chinese Academy of Sciences, Guangzhou, China; ^2^Southern Marine Science and Engineering Guangdong Laboratory, Guangzhou, China; ^3^Graduate School of Agriculture, Hokkaido University, Sapporo, Japan; ^4^Sarawak Tropical Peat Research Institute, Kota Samarahan, Malaysia; ^5^Department of Environmental and Biological Sciences, University of Eastern Finland, Joensuu, Finland

**Keywords:** *Sphagnum* moss, bacteria, N_2_O emitters, N_2_O-related genes, pH, permafrost peat

## Abstract

Melting permafrost mounds in subarctic palsa mires are thawing under climate warming and have become a substantial source of N_2_O emissions. However, mechanistic insights into the permafrost thaw-induced N_2_O emissions in these unique habitats remain elusive. We demonstrated that N_2_O emission potential in palsa bogs was driven by the bacterial residents of two dominant *Sphagnum* mosses especially of *Sphagnum capillifolium* (SC) in the subarctic palsa bog, which responded to endogenous and exogenous *Sphagnum* factors such as secondary metabolites, nitrogen and carbon sources, temperature, and pH. SC's high N_2_O emission activity was linked with two classes of distinctive hyperactive N_2_O emitters, including *Pseudomonas* sp. and *Enterobacteriaceae* bacteria, whose hyperactive N_2_O emitting capability was characterized to be dominantly pH-responsive. As the *nosZ* gene-harboring emitter, *Pseudomonas* sp. SC-H2 reached a high level of N_2_O emissions that increased significantly with increasing pH. For emitters lacking the *nosZ* gene, *an Enterobacteriaceae* bacterium SC-L1 was more adaptive to natural acidic conditions, and N_2_O emissions also increased with pH. Our study revealed previously unknown hyperactive N_2_O emitters in *Sphagnum capillifolium* found in melting palsa mound environments, and provided novel insights into SC-associated N_2_O emissions.

## Introduction

Arctic permafrost soils store ample nitrogen (N) reservoirs that may be subject to remobilization due to climate warming (Christensen et al., 2013), that leads to permafrost degradation and thawing (Borge et al., 2017). After permafrost thaws, increased nitrous oxide (N_2_O) emissions are observed in arctic permafrost peatlands (Voigt et al., 2017a,b). N_2_O is a potent greenhouse gas and contributes to the disruption of the ozone layer (IPCC, 2007; Ravishankara et al., 2009). Therefore, urgency to understand the primary source of N_2_O emissions in this arctic environment is crucial.

Peatlands store one-third of global soil carbon, and boreal peatlands account for 83% of the global peatland area (Eurola et al., 1984; Savolainen et al., 1994). Bare peat in permafrost peatlands has been identified as a hot spot for N_2_O emissions due to low availability nitrogen (N) competition in subarctic tundra (Repo et al., 2009; Marushchak et al., 2011). *Sphagnum*-dominated bogs have low nutrient content, low primary production, low-quality plant litter, low litter decomposition rates, and low mineral content combined with a low pH (<4.5) environment, which is vital for carbon (C) sequestration (Chronáková et al., 2019). Mineral N deposition to *Sphagnum* bogs has progressed, with ammonification, ammonia oxidation, and denitrification playing a critical role in the emission of N_2_O (Van Cleemput, 1998; Francis et al., 2007). In addition, the water table level also affects N_2_O emissions in northern peatland, as lowering the water table leads to increased N_2_O production (Regina et al., 1996). Once the peatlands are drained, *Sphagnum* vegetation and surface peat layers are exposed to the atmosphere, activating nitrification due to ammonium (${\text{NH}}_{4}^{+}$-N) release in aerobic peat degradation, followed by denitrifier stimulation in N-enriched conditions to emit N_2_O (Martikainen et al., 1995; Regina et al., 1999; Minkkinen et al., 2020). Palmer and Horn (2012) reported that palsa peatlands in the northwestern Finnish Lapland showed N_2_O emissions *in situ* from −0.02 to 0.01 μmol N_2_O m^−2^ h^−1^. Emissions of N_2_O may rise considerably during the thaw of permafrost, representing another ongoing change in northern peatlands. It was reported that a five-fold increase in N_2_O flux from palsa mire peat in a permafrost thaw experiment (Voigt et al., 2017b). However, determining which active N_2_O emitters in these northern ecosystems contribute to high emissions remains largely elusive.

*Sphagnum* mosses (non-vascular plants) dominate the vegetation of many northern mire ecosystems and harbor a high diversity of nitrifiers and denitrifiers (Dedysh et al., 2006; Gilbert et al., 2006; Opelt et al., 2007). In these moss communities, N_2_O gas is mainly produced *via* nitrification, nitrifier denitrification, and denitrification pathways (Wrage et al., 2001). High hummocks in bogs and palsa mire permafrost mounds have relatively thick aerobic acrotelm layers and are the most potential microhabitats to N_2_O emissions. These microhabitats are characteristically dominated by *Sphagnum fuscum* (SF) and *Sphagnum capillifolium* (SC) (Markham, 2009; Novak et al., 2015; Zhong et al., 2020), which are widely distributed throughout European and North American peat bogs. These keystone species develop climax-type, raised bog hummock vegetation. Upon exposure to high N inputs, polyphenol secondary metabolites produced by these *Sphagnum* mosses, such as caffeic acid, are often reduced (Bragazza and Freeman, 2007; Montenegro et al., 2009). These secondary metabolites may impact the activity and community composition of the microbiota within the holobiont and the associated N_2_O emission rates (Wang and Cernava, 2020).

Our previous work has demonstrated that the N_2_O source in southeastern Finland was mainly from *Sphagnum* moss rather than peat soil. However, this previous study only focused on the single keystone and dominant species of SF in Finnish temperate marine climate areas (Nie et al., 2015). The different contributions of N_2_O emissions between several dominant *Sphagnum* species, especially in a typical subarctic permafrost peatland [hot-spots of N_2_O emission (Voigt et al., 2017b)] in Finland, is largely unknown. This study uses SF as the control plants and aim to answer three questions: (1) Are the N_2_O emission potentials between the two dominant *Sphagnum* species (SC and SF) similar or different in the subarctic palsa bog? (2) How does the culture-based N_2_O assay for the bacterial community composition of the two *Sphagnum* species influence the N_2_O emission potential? (3) What is the dominant process of N_2_O production by active N_2_O emitters under aerobic conditions of peat bogs? By investigating N_2_O emission potential in SF and SC grown in drained palsa peat bogs of northwestern Finland, we aim to characterize the dominant N_2_O emitters hidden in the microbiota of SF and SC in association with their N_2_O emission traits in response to major holobiont factors.

## Materials and methods

### Sampling *Sphagnum* mosses

**Figure 1:**
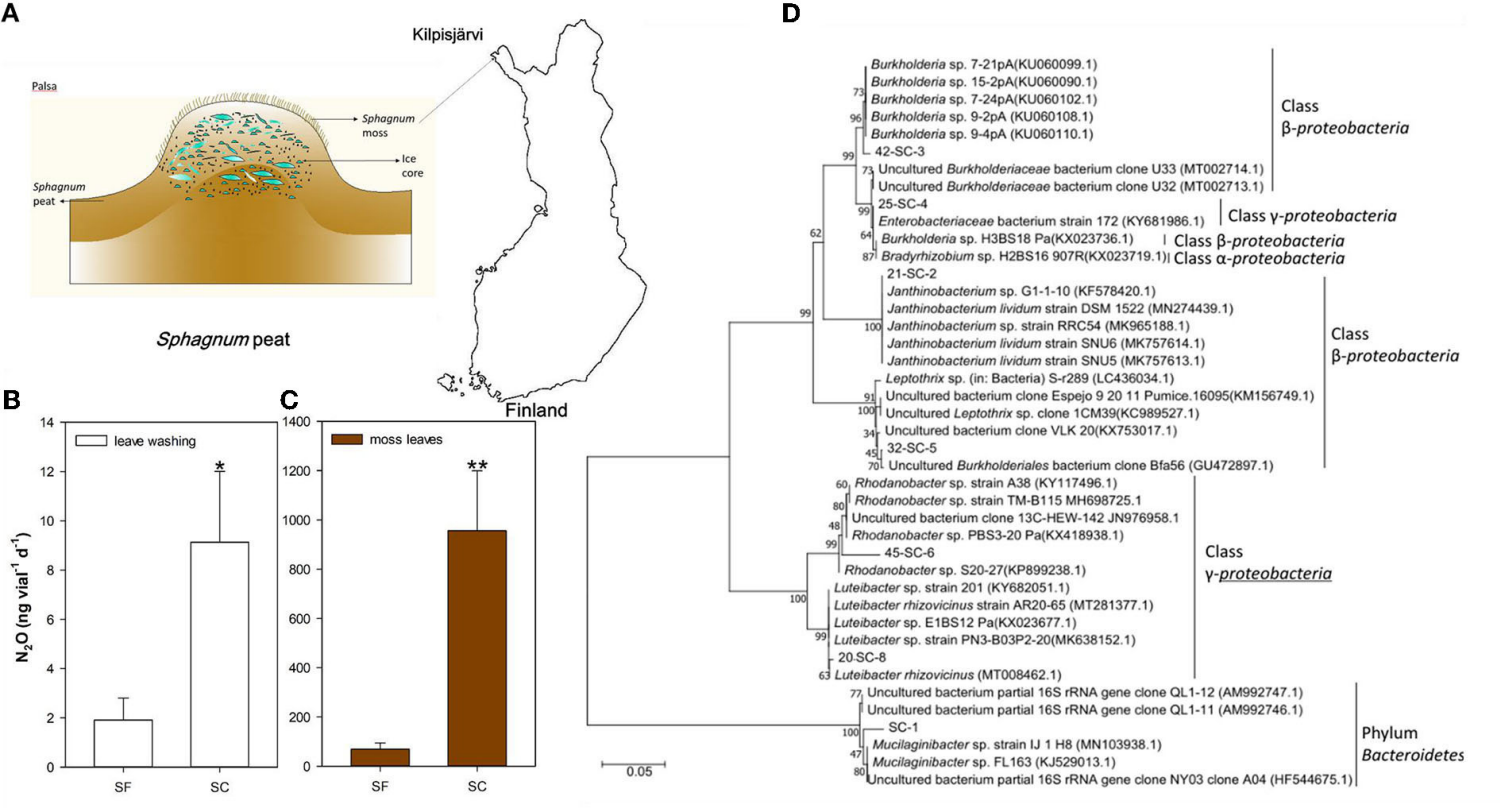
N_2_O emission potential and microbial communities in the *Sphagnum* mosses grown in Finland's plateau of a permafrost mound. Sample site of *Sphagnum* mosses **(A)**. 100 μL *Sphagnum* leaves washing (100 mg/10 ml) as inoculants **(B)**. *Sphagnum* mosses as the inoculants **(C)**. The community structure of bacteria revealed by the PCR-DGGE profile was subjected to phylogenetic analysis of SC **(D)**. Incubation conditions of **(B,C)**: pH = 5, incubated at 15°C, 7 days, *n* = 3, with 0.05% sucrose. *P< *0.05 and* **P< *0.01*.

Composite samples of SF and SC (photos of them at one site are shown in Supplementary Figure S1) were collected from a plateau of a permafrost mound of a palsa mire near Kilpisjärvi (68°43′; 21°25′), northwestern Finland (Figure 1A). Each sample of SC/SF was formed from three random sampling sites with three replicates in August–September, 2014. SC and SF were collected from the same patch (within 50–100 cm) and the sampling sites were 50 to 100 m away from each other. From each sampling site, random 533 to 565 individual plants of either SC or SF were collected and mixed for each sample in order to guarantee the sample's representation. Both SC and SF were collected from large homogenous stands with a 40 cm thaw layer above the permafrost surface. The region has a low annual mean temperature (−2.3°C) and moderate mean annual precipitation (487 mm). The growing season is one of the shortest in continental Europe (~100 d when the mean daily temperature is ≥5°C). The *Sphagnum* samples stored in Ziploc® bags at 4°C were used for further culture-based N_2_O emission measurements.

### Comparison of N_2_O emission potentials in two *Sphagnum* mosses

To evaluate the potential for N_2_O emission of the two *Sphagnum* mosses under an experimental nitrogen load, we took three *Sphagnum* mosses plants (~0.1 g in dry weight) randomly from the respectively, composite sample of SC and SF using sterilized tweezers. At the same time, we standardized the dry weight for the N_2_O assay. Either 100 μL of *Sphagnum* moss leaf extract (100 mg/10 ml) or 3 plants were added to N_2_O assay medium [10 ml of modified Winogradsky's Gellan (MWG) medium containing 0.005% yeast extract and solidified with 3% gellan gum with 22.6 ml of headspace in each vial (30 ml gas-chromatographic vial with a butyl rubber plug) (Nichiden-Rika Glass Co., Kobe, Japan)] with 0.05% sucrose diluted with sterilized Milli-Q water (the solution was adjusted to pH = 5.0 with 2 M H_2_SO_4_) (three replicates in each case) (Hashidoko et al., 2008). After incubation at 15°C (according to the mean value of summer temperature of Finland) for 7 days in the dark, an N_2_O assay was carried out by using an electron capture detector(ECD)-gas chromatograph (Shimadzu GC-14B, 125 Kyoto, Japan) connected to a Porapak N column (1 m long, Waters, Milford, MS, USA). In another treatment, 0.1 g L^−1^ of caffeic acid instead of 0.05% sucrose was added as the carbon source to the vials with three plants (~0.1 g in dry weight) randomly taken from the above composite samples (pH 5). A control for the assay, without any carbon source, was also performed simultaneously (three replicates in each case). After incubation at 15°C in the dark for 4, 8, and 15 days, an assay of N_2_O was performed as mentioned above.

### DGGE profiling of the bacterial communities in two *Sphagnum* species

Polymerase chain reaction-denatured gradient gel electrophoresis (PCR-DGGE) was performed to observe the culture-based bacterial communities on the leaves of the two *Sphagnum* mosses. First, genomic DNA was extracted from the medium after the N_2_O assay using an Isoplant II DNA Extraction kit (Nippon Gene, Toyama, Japan). The PCR steps and conditions were as follows: PCR denaturation for 5 min at 95°C, and 30 cycles of amplification (15 s at 95°C, 30 s at 55°C, 30 s at 72°C), and 10 min elongation at 72°C. Then PCR products for DGGE were obtained by using the common 16S rRNA primers GC-341F (CGC CCG CCG CGC CCC GCG GGG GTC CCG CCG CCC CCG CCC GCC T AC GGG AGG CAG CAG) and 907R (CCG TCA ATT CCT TTR AGT TT) (Ferris et al., 1996) and run on a 30–70% denatured gradient gel (6% w/v). The sequences of DGGE-cutting bands were obtained using an ABI prism^TM^ 310 Genetic Analyzer and retained in the NCBI (BioProject No. PRJNA681491).

### Culture-dependent screening and identification of N_2_O emitters

100 μl of medium with three *Sphagnum* mosses (after incubation for 7 days) was diluted 1× 10^4^- and 10^6^-Fold and inoculated onto MWG plates to screen N_2_O emitters. After incubation for 5 days at 20°C in the dark, 13 distinguishable bacterial colonies characterized by colony characteristics were selected for streak cultivation on MWG plates and transferred to potato dextrose agar (PDA) plates until purified. Each Pure strain [a total of 108 isolates (13 bacterial colonies with 8 replicates), with 100 μl of each bacterial cell suspension (OD_660nm_ = 0.9–1.0)] was inoculated into an N_2_O assay vial with 10 ml of modified MWG medium to test their N_2_O emission ability. The three pure strains SC-K1, SC-L1, and SC-H2 (from SC) showed relatively higher N_2_O production and were active N_2_O emitters (Supplementary Table S2, data collected from six top active N_2_O emission-bacterial colonies). The genomic DNA of each strain was extracted, and the 16S RRNA gene was amplified through PCR by using a series of primers 27F, 338R, 341F, 907R, 1080R, 1380R, 1492R, 1112F, and 1525R. Sequencing was performed with an ABI Prism^TM^ 310 Genetic Analyzer (Applied Biosystems, USA) (Nie et al., 2015). All the resulting 16S RRNA gene sequencing datasets were deposited in the NCBI database (accession nos. MW301596–MW301598) and compared with sequences in the nucleotide basic local alignment search tool (BLASTN) database program provided by NCBI (National Center of Biotechnology Information, Bethesda, MD, USA; http://Blast.Ncbi.nlm.nih.gov/Blast.cgi).

### N_2_O emitters response to nitrogen sources, pH, and temperature

The pure isolates (SC-K1, SC-L1, and SC-H2) pre-cultured on PDA for 4 days at 15°C were separately scraped with a nichrome wire loop and suspended into 1.5 ml Milli-Q water (equal amounts of each pure strain was guaranteed). A 20 μl portion of the inoculant that showed an optical density of OD_660nm_ 0.9–1.0 was added to the N_2_O assay vial and then was thoroughly vortexed for 30s. 1 mM NH_4_NO_3_, KNO_3_, and NH_4_Cl were tested and incubated at 15°C for 5 days with 0.05% sucrose (pH = 5.0) to determine the optimal nitrogen substrates for pure N_2_O emitters. The pH was adjusted with 1 M H_2_SO_4_ and 1 M KOH solutions to 4.6, 5.0, 5.7, 6.8, and 7.3 before autoclaving and incubated at 15°C for 5 days with 0.05% sucrose to determine the optimal pH for N_2_O emitters. Different temperatures (4, 10, 15, 20, 25, and 30°C) were set in separate incubators and incubated for 5 days with 0.05% sucrose to find the appropriate temperature. All experiments were performed with three replicates.

### Carbon source- and polyphenol-supplementation assays

Sucrose and *E-*caffeic acid were applied as carbon sources and secondary metabolites (polyphenols), respectively, for the microbiota inhabiting *Sphagnum* moss (Nie et al., 2015). The inoculants were prepared as described in Nie et al. (2015). To observe the responses of the N_2_O emitters (SC-K1, SC-L1, SC-H2) to sucrose, 0 (control), 0.05, and 0.5% sucrose were used for the separated/cultivated bacterial strains. To determine the optimal concentrations of *E-*caffeic acid for N_2_O emitters (SC-K1, SC-L1, SC-H2), 0 (control), 0.005, 0.01, 0.05, 0.1, 0.5, and 1 g L^−1^ *E-*caffeic acid were used. Each treatment contained three analytical replicates incubated at 15°C for 5 days with inoculants for N_2_O assays. Their N_2_O emissions were separately measured.

### Analysis of denitrification rates of N_2_O emitters

We applied the acetylene inhibition assay, which is widely used to measure denitrification rates (Sørensen, 1978). The activity of N_2_O reductase was inhibited by adding acetylene (C_2_H_2_) at pH 5.0 and 7.0, and 10% C_2_H_2_ gas was injected into the headspace of vials inoculated with N_2_O emitters (the same with above inoculation method) (Bollmann and Conrad, 1997). At the same time, treatments without injected C_2_H_2_ gas were carried out as controls to compare the N_2_O reductase activity (three replicates in each case). Incubation conditions were the same as described above.

### Detection of nitrogen cycling functional genes in N_2_O emitters

Functional genes of nitrogen cycling, including *narG, nirK, nirS*, and *nosZ* (Supplementary Figure S4), were detected by using the PCR method. The target genes were amplified by using the primers *narG*F (TCG GGC AAG GGC CAT GAG TAC) and *narG*R (TTT CGT ACC AGG TGG CGG TCG), *nirS*Cd3Af (AAC GYS AAG GAR ACS GG) (Nie et al., 2015) and *nirS*R3cd (GAS TTC GGR TGS GTC T) (Throbäck et al., 2004), *nirK*-1F (GGM ATG GTK CCS TGG CA) and *nirK*-5R (GCC TCG ATC AGR TTR TGG) (Braker et al., 1998), *nosZ*-1111F (STA CAA CWC GGA RAA SG), *nosZ*-661F (CGG CTG GGG GCT GAC CAA), *nosZ*-1527R (CTG RCT GTC GAD GAA CAG), and *nosZ*-1773R (ATR TCG ATC ARC TGB TCG TT) (Scala and Kerkhof, 1998). The exact reaction conditions of the PCR amplifications are presented in Supplementary Table S1.

### Statistical analysis

The data were expressed as mean with standard error (SE). The data were examined for normality and homoscedasticity using the Shapiro-Wilk's and Levene's tests, respectively (SPSS, version 23.0). All data was found to fit the normal distribution and homogeneity of variances. Comparisons were made using a one-way analysis of variance (ANOVA) among two or more groups. One-way ANOVA was used to compare differences in N_2_O emission with different inoculants (*Sphagnum mosses* or their leaves washing), physicochemical factors [pH, temperature, sucrose, nitrogen types, and secondary metabolite (*E*-caffeic acid)], and C_2_H_2_ inhibition assay. Using the Fisher's Least Significant Difference(LSD) method, multiple comparisons were carried out using IBM SPSS 23.0 software (Chicago, Illinois, USA).

## Results

### N_2_O emission potential and microbial communities

After incubation for 7 days, we found that the average N_2_O emissions of SF were 1.9 ng vial^−1^ d^−1^ in the leaf extract and 69.9 ng vial^−1^ d^−1^ in the leaf samples. The SC sample showed N_2_O emissions of 9.1 in the leaf extract and 956.2 ng vial^−1^ d^−1^ in the leaf samples (Figures 1B,C).

**Figure 2:**
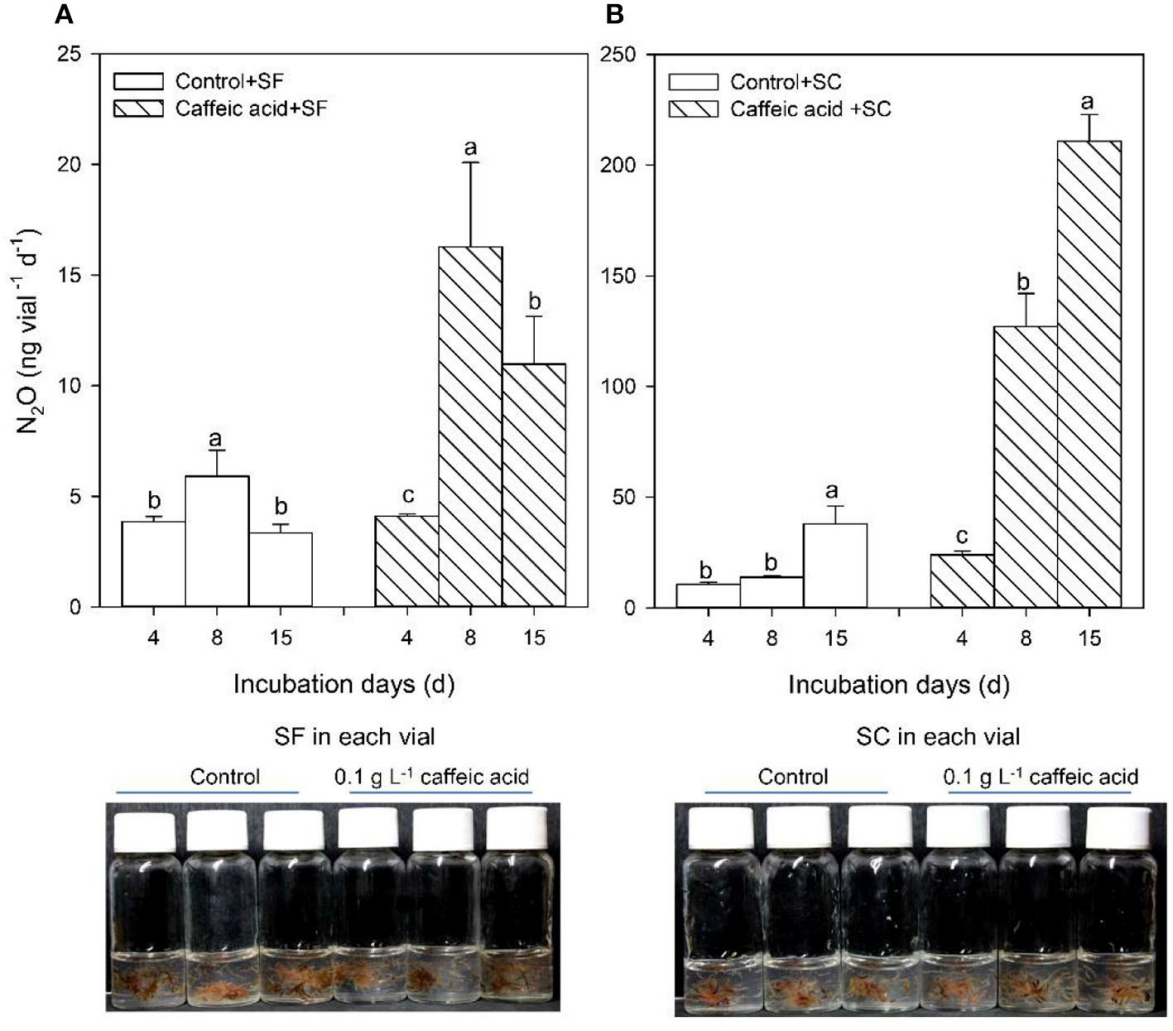
Response of N_2_O production of *Sphagnum fuscum* (SF) and *Sphagnum capillifolium* (SC) to caffeic acid. Response of N_2_O production of *Sphagnum fuscum* (SF) to caffeic acid **(A)**. Response of N_2_O production of *Sphagnum capillifolium* (SC) to caffeic acid **(B)**. Incubation conditions: pH = 5, incubated at 15°C, 4, 8, 15 days, *n* = 3, without sucrose. Values are means ± s.e. (shown as error bars).

The PCR-DGGE profile showed that the major culture-based bacterial communities in these *Sphagnum* mosses were similar. However, the SC sample harbored the family *Enterobacteriaceae* (Figure 1D, Supplementary Figure S2), while the SF sample contained the genus *Dyella* of Gammaproteobacteria (Supplementary Figure S2). N_2_O production increased with 0.1 g L^−1^ caffeic acid addition in both samples, and the effect was significantly larger in the SC sample than in the SF sample (*p* < 0.05) (Figures 2A,B).

### Major N_2_O emitters in *Sphagnum* mosses

Compared to PCR-DGGE, the culture-based approach revealed distinctive profiles of N_2_O emitters (Supplementary Figure S2). Two *Burkholderia* spp. were isolated from the SF sample, while three *Gammaproteobacteria* (one *Pseudomonas* sp., one *Serratia* sp., and an unidentified *Enterobacteriaceae* bacterium) and one *Burkholderia* sp. were isolated from the SC sample. Among them, *Serratia* sp. SC-K1, *Enterobacteriaceae* bacterium SC-L1, and *Pseudomonas* sp. SC-H2 showed the most efficient N_2_O emissions, and the activity of N_2_O emissions was the greatest in *Pseudomonas* sp. SC-H2, then *Enterobacteriaceae* bacterium SC-L1, and then *Serratia* sp. SC-K1 (pH 5) (Table 1, Supplementary Table S2).

**Table 1:** Identification of the active N_2_O emitters using 16s rRNA gene sequence.

**Isolates**	**Length (bp)**	**Accession No**.	**Most aligned DNA (Accession No.)**	**Identities**
SC-K1	1528	MW301598	*Serratia* sp. HC3-14(JF312984.1)	1515/1526(99%)
			*Serratia* sp. HC3-9(JF312979.1)	1513/1525(99%)
			*Serratia* sp. HC4-9(JF312995.1)	1512/1525(99%)
SC-L1	1165	MW301597	*Serratia liquefaciens* strain Noth_10 (MF716557.1)	1123/1153(97%)
			*Enterobacteriaceae* bacterium ENUB8 (JX162036.1)	1133/1167(97%)
			*Serratia proteamaculans* strain 336X(CP045913.1)	1132/1167(97%)
SC-H2	1514	MW301596	*Pseudomonas* sp. LH1G9(CP026880.1)	1513/1518(99%)
			*Pseudomonas* sp. 05CF15-5C (LC007966.1)	1513/1518(99%)
			*Pseudomonas* sp. Pi 3-62 (AB365063.1)	1512/1517(99%)

### Effects of substrate type, temperature and pH on microbial N_2_O emissions

**Figure 3:**
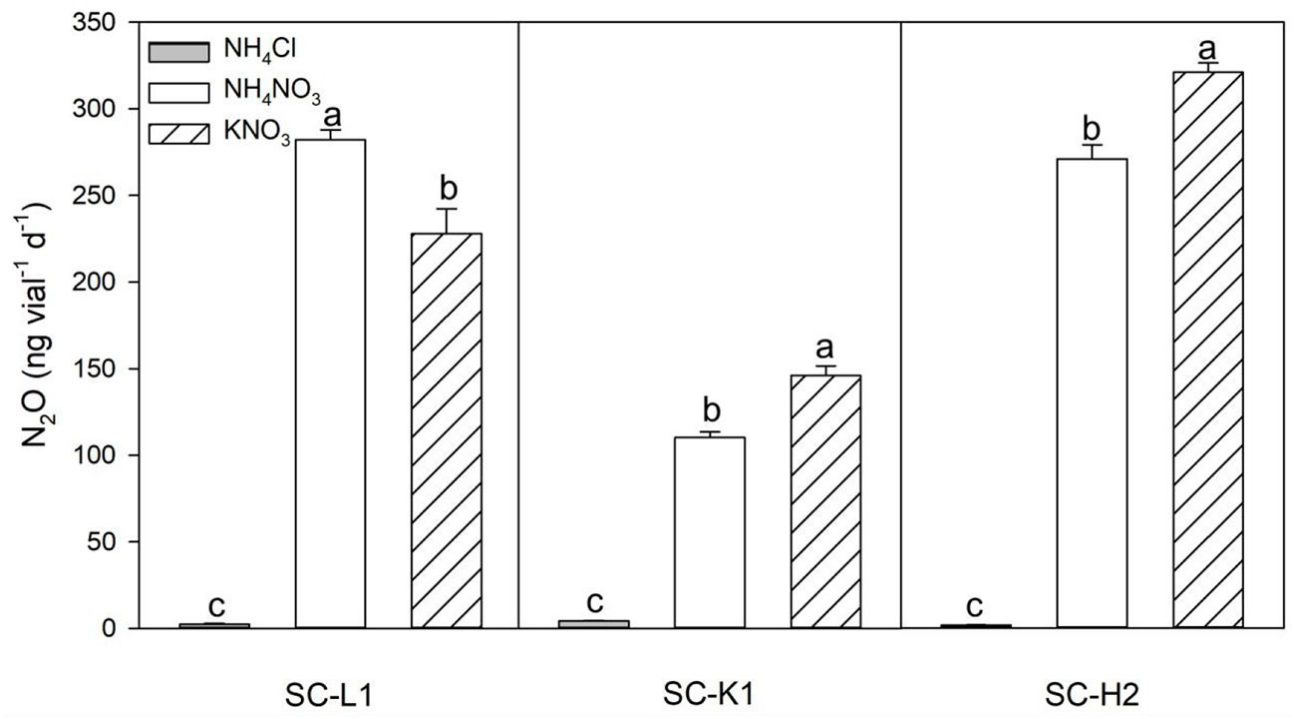
N_2_O emission by three pure N_2_O emitters (SC-L1, SC-K1, SC-H2) upon exposure to different nitrogen substrates (1 mM NH_4_Cl, NH_4_NO_3_, KNO_3_). Incubation conditions: pH = 5, incubated at 15°C for 5 days with 0.05% sucrose (*n* = 3). Values are means ± s.d. (shown as error bars).

**Figure 4:**
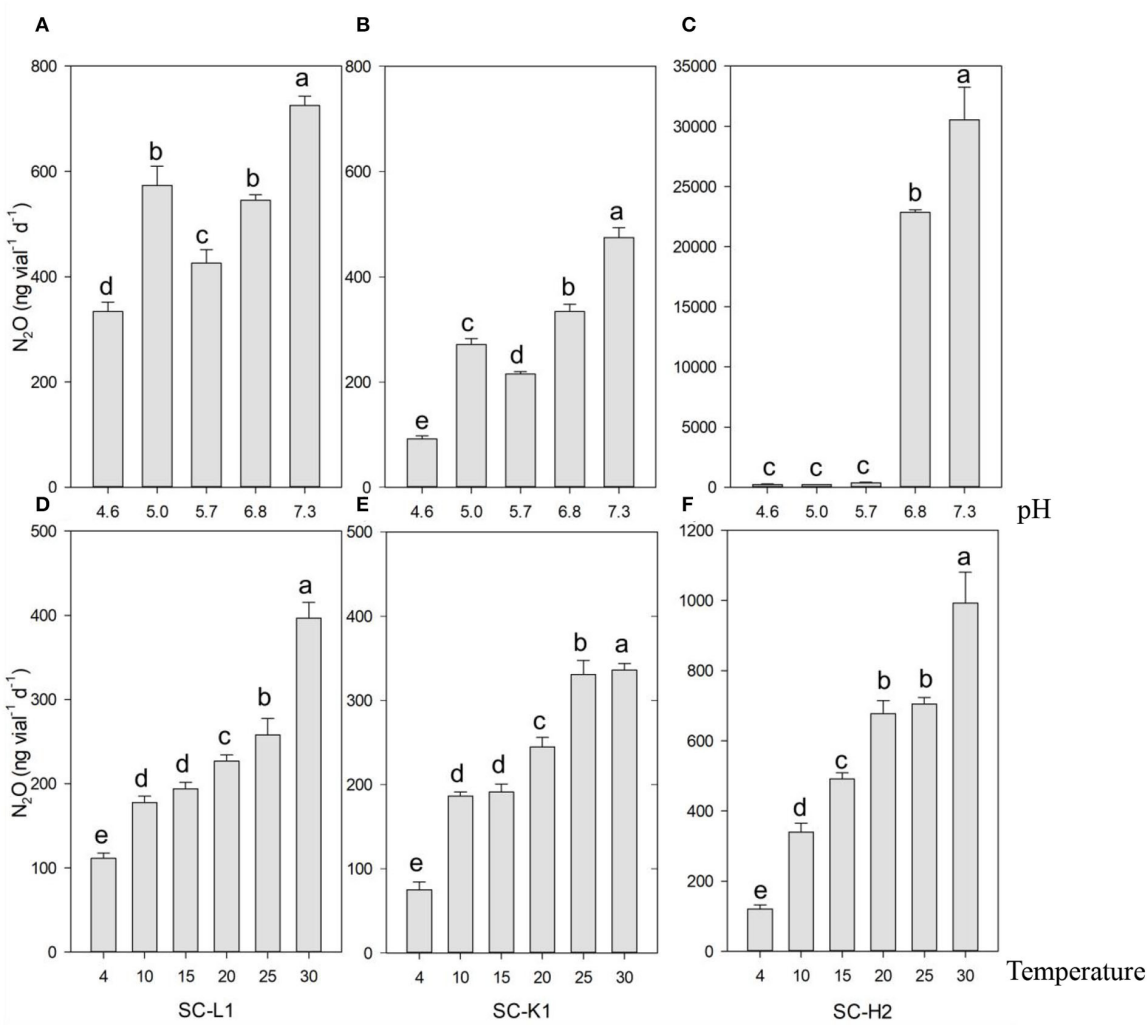
N_2_O emission by three pure N_2_O emitters (SC-L1, SC-K1, SC-H2) upon the gradient pH and temperature. N_2_O emission by SC-L1 **(A,D)**, SC-K1 **(B,E)**, SC-H2 **(C,F)** upon exposure to different pH from 4.6 to 7.3 **(A–C)**, and different temperatures from 4 to 30 °C **(D–F)** was analyzed. For the impact of pH on N_2_O emission, the N_2_O emitters were incubated at 15°C for 5 days with 0.05% sucrose (*n* = 3). For the impact of temperature on N_2_O, the N_2_O emitters were incubated for 5 days with 0.05% sucrose (*n* = 3 and pH = 5).

According to the N_2_O production responses to different nitrogen sources, KNO_3_ was the most efficient substrate for N_2_O emission, followed by NH_4_NO_3_, while almost no N_2_O emissions were found with NH_4_Cl as the substrate. Active N_2_O emissions from KNO_3_ indicated that the three N_2_O emitters were nitrate reducers (Figure 3). N_2_O emissions increased as the pH increased from 4.6 to 7.3. *Enterobacteriaceae* bacterium SC-L1 and *Serratia* sp. SC-K1 showed a temporary increase at a pH value of 5 but no drastic increase in N_2_O emissions, indicating adaptation to acidic environments (Figures 4A,B). At pH values over 6, *Pseudomonas* sp. SC-H2 emissions increased sharply, making it the most likely N_2_O emitter (Figure 4C). For the three strains used, N_2_O emissions also increased with increasing temperature from 4 to 30°C (Figures 4D–F).

### Disparate responses of N_2_O emitters to caffeic acid and sucrose

**Figure 5:**
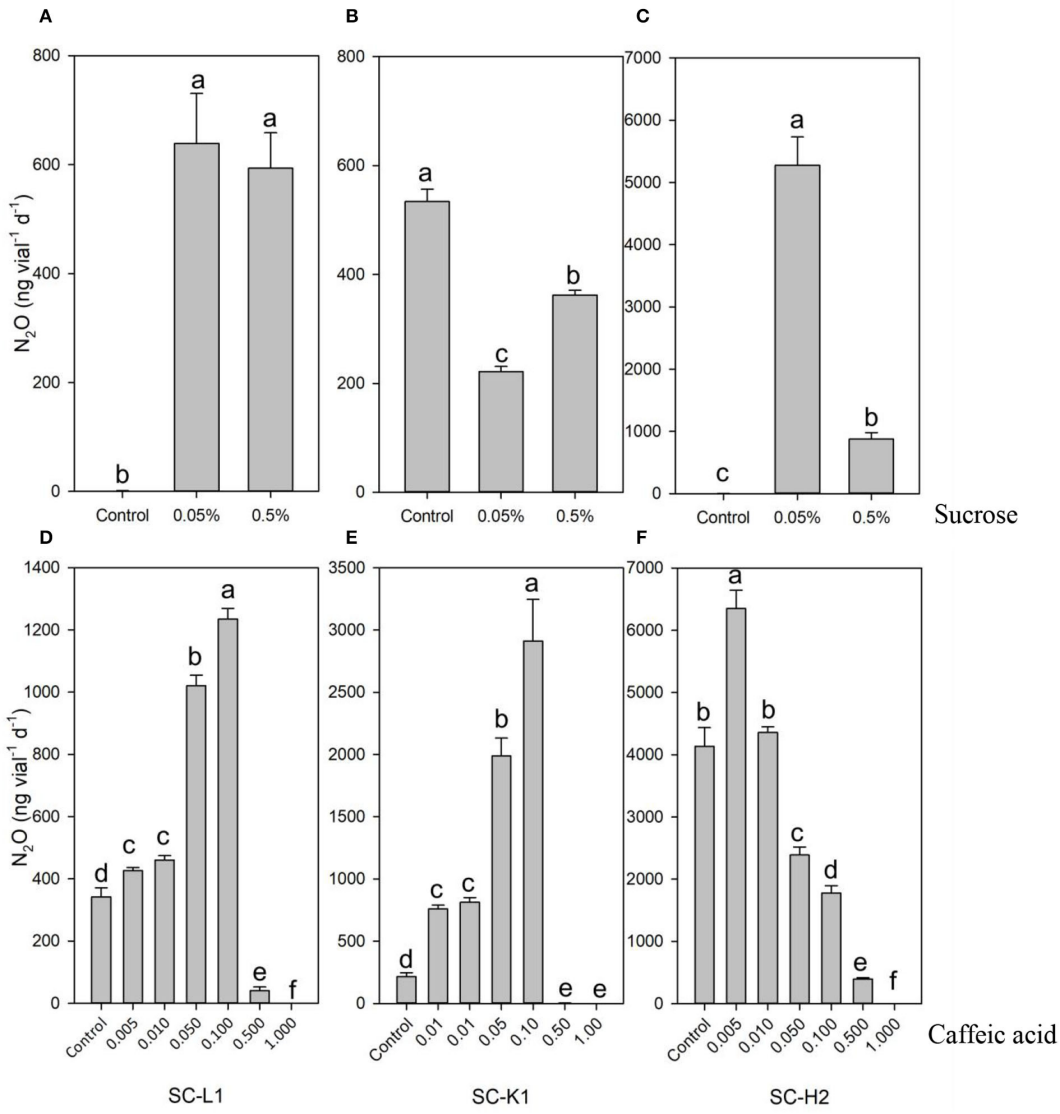
**(A)** N_2_O emission by three pure N_2_O emitters (SC-L1, SC-K1, SC-H2) exposure to the different concentrations of sucrose **(A–C)** and caffeic acid **(D–F)**. N_2_O emission by SC-L1 **(A,D)**, SC-K1 **(B,E)**, SC-H2 **(C,F)** upon exposure to different concentration of sucrose from 0 to 0.5% **(A–C)** and different concentration of caffeic acid from 0 to 0.1 g L^−1^ **(D–F)** was analyzed. For the impact of sucrose on N_2_O emission, the N_2_O emitters were incubated at pH = 7 for 5 days (*n* = 3), and the control was without supplemented sucrose. For the impact of caffeic acid on N_2_O emission, the N_2_O emitters were incubated at pH = 7 for 5 days with 0.05% sucrose (*n* = 3), and the control was without supplemented caffeic acid.

The three microbial strains exhibited disparate responses to sucrose and *E-*caffeic acid (Figure 5). In the absence of added sucrose (control treatment), *Serratia* sp. SC-K1 emitted more N_2_O than *Enterobacteriaceae* bacterium SC-L1 and *Pseudomonas* sp. SC-H2, while these last two strains emitted N_2_O at higher levels with 0.05% sucrose supplementation (Figures 5A,B). Notably, the response of *Pseudomonas* sp. SC-H2 to 0.05% sucrose was very drastic, resulting in emission ~2x10^3^ times higher than without sucrose (Figure 5C). This result demonstrated that *Serratia* sp. SC-K1 is an oligotrophic bacterium, whereas *Enterobacteriaceae* bacterium SC-L1 and *Pseudomonas* sp. SC-H2 are eutrophic bacteria.

For the pure strains of *Enterobacteriaceae* bacterium SC-L1 and SC-K1, a relatively lower concentration of *E-*caffeic acid ( ≤ 0.1 g L^−1^) increased N_2_O emissions of these two strains, and the optimum concentration was 0.1 g L^−1^ (Figures 5D,E). Among them, *Serratia* sp. SC-K1 was very sensitive to 0.1 g L^−1^, and 13-fold higher N_2_O production was found than without *E-*caffeic acid (Figure 5E). For *Pseudomonas* sp. SC-H2, when the concentration of *E-*caffeic acid was above 0.01 g L^−1^, N_2_O emissions decreased significantly (*p* < 0.01) (Figure 5F).

### Modest responses of N_2_O emitters to acetylene

**Figure 6:**
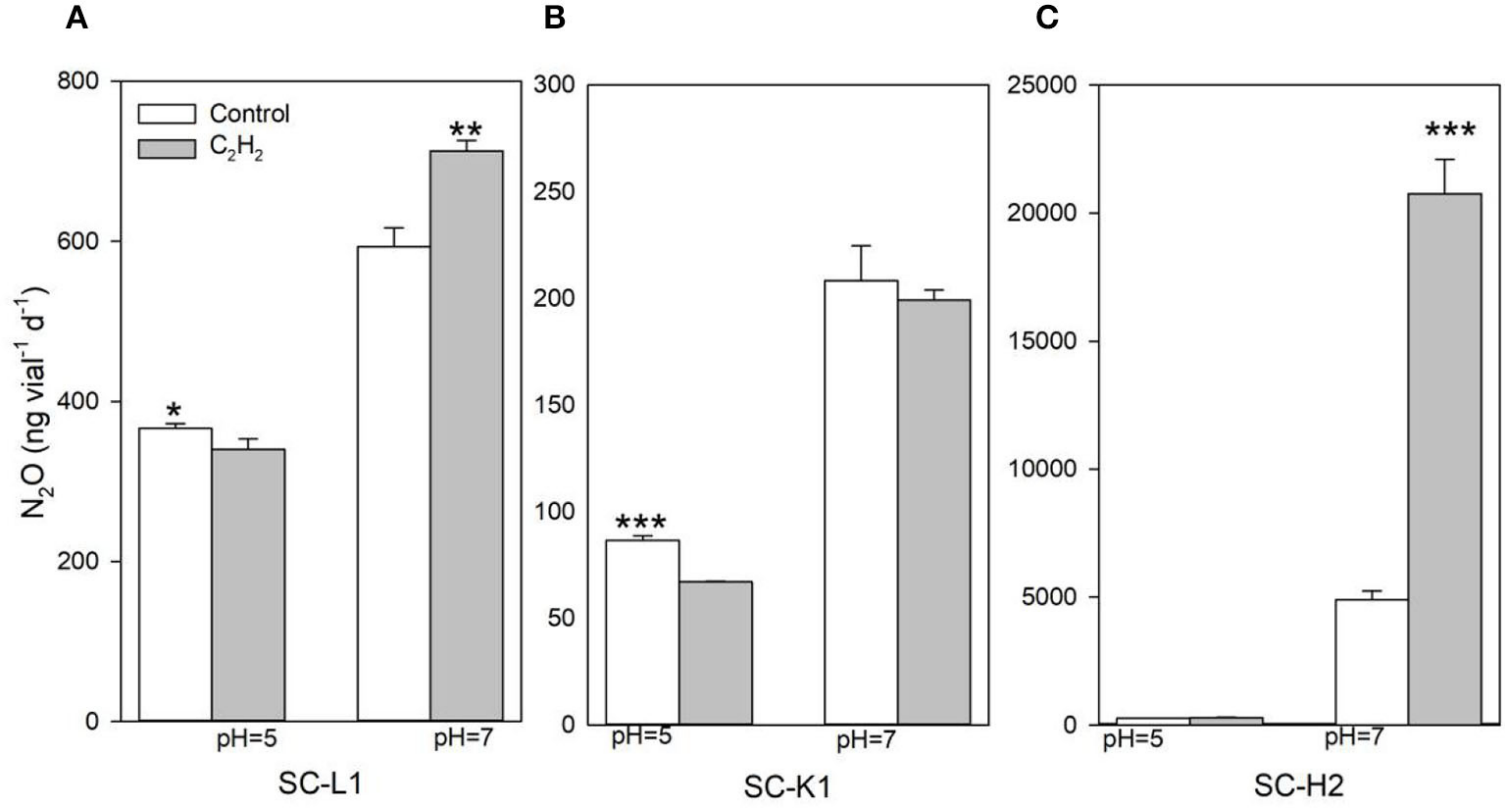
N_2_O emission by three N_2_O emitters (SC-L1, SC-K1, SC-H2) exposure to 10% C_2_H_2_ gas **(A–C)**. The three N_2_O emitters were incubated at pH 5 and 7, under 15°C with 0.05% sucrose for 5 days (*n* = 3). Without C_2_H_2_ gas was used as a control. *P< *0.05*, **P< *0.01, and* ***P< *0.001*.

There was no detectable difference between the 10% C_2_H_2_ and control treatment emissions at a pH value of 5.0. However, in *Pseudomonas* sp. SC-H2 cultured at a pH value of 7.0, N_2_O emissions upon exposure to C_2_H_2_ were drastically increased to four-fold higher than that of the control. Without 10% C_2_H_2_, the production level of N_2_O at a pH value of 7.0 was higher than that at a pH value of 5.0 (Figure 6). This result suggested that the peat ecosystem was highly disturbed at a pH value of 7.0, denitrification was greatly accelerated, and the final denitrification step to reduce N_2_O to N_2_ was driven by N_2_O reductase.

### Functional genes involved in N_2_O emission

PCR assays detected the *narG* gene in the three N_2_O emitter strains, but only *Pseudomonas* sp. SC-H2 contained *nirS* and *nosZ* genes (Table 2; Supplementary Figure S3). In combination with the results of the C_2_H_2_ assay, these results suggested that *Pseudomonas* sp. SC-H2 is a complete denitrifier. The *nir*K gene was not detected within *Enterobacteriaceae* bacterium SC-L1 and *Serratia* sp. SC-K1.

**Table 2:** Characteristics of the three active N_2_O emitters isolated from SC and PCR assay to detect denitrification-related genes.

**Isolates**	**Optimal pH**	**Optimal**	**Optimal**	**Sucrose %)**	***E*-caffeic acid**	***nar*G**	***nir*S**	***nir*K**	***nosZ***
		**temperature (°C)**	**substrates**		**(g L^−1^)**	
SC-L1	7.3	30	NH_4_NO_3_	0.05/0.5	0.1	+	–	–	–
SC-K1	7.3	30	KNO_3_	0	0.1	+	–	–	–
SC-H2	7.3	30	KNO_3_	0.05	0.005	+	+	–	+

−; indicated the isolates without the functional genes. +; indicated the isolates harboring the functional genes.

## Discussion

### Cultured bacterial communities in the leaves distinguishable between two *Sphagnum* species

Increased atmospheric N deposition can reduce the growth of some *Sphagnum* species, such as *Sphagnum magellanicum* (Aerts et al., 2001; Limpens and Berendse, 2003). In contrast, the production of SF increased with elevated N deposition but decreased as N deposition reached 14.0 kg ha^−1^ yr^−1^ as reported by Vitt et al. (2003). SC can also tolerate a high N supply (Bonnett et al., 2010). Our study offered evidence that individual samples of the latter two *Sphagnum* species had N_2_O emission potential reasonably associated with their bacterial communities. In particular, the SC sample harbored specific bacterial communities associated with high N_2_O emission. Surprisingly, the N_2_O emission of the SC sample was significantly greater than that of the SF sample (Figure 1B) (*p* < 0.01). Such a large difference in N_2_O emission between the SF and SC species gives precedence to the hypothesis of potential N_2_O emission differences in different *Sphagnum* species.

Based on the analysis of bacterial communities using culture-based PCR-DGGE and isolation of N_2_O emitters, the major *Sphagnum*-associated bacterial communities of our samples were consistent with boreal mire and tropical peat forest and included *Burkholderia, Mucilaginibacter, Rhodanobacter*, and *Janthinobacterium* but their N_2_O emission activity was different in varied sites due to differences in climate and habitat environments (Hashidoko et al., 2008; Sun et al., 2014). *Janthinobacterium* spp. did not show high N_2_O emission potential in subarctic palsa bog unlike in the tropical peatland soil, which suggested that the N_2_O emission functions of N_2_O emitters were changing in different climate zones. Previous experimentation has shown that the *Sphagnum* microbiota supported the host plant and the entire ecosystem under environmental changes (Bragina et al., 2014). *Burkholderia* spp. were N_2_O emitters, but their N_2_O emission functions were significantly lower than the acid-tolerant *Janthinobacterium* sp. in a deforested tropical peatland soil, which was previously determined by soil pH (Hashidoko et al., 2010). The *Burkholderia* spp. isolates in SF were similar to another climate zone in Finland, showing the same species of *Sphagnum* although in a different climate zone (Nie et al., 2015). Within this study, some unique bacterial strains were found in the leaves of SC, including a *Pseudomonas* sp. and two *Enterobacteriaceae* family members. In numerous previous studies, *Pseudomonas* species (*P. denitrificans, P. perfectomarinus, P. fluorescens, P. stutzeri, P. aeruginosa*, and *P. nautica*) were found performing denitrification (Delwiche, 1959; Payne et al., 1971; Balderston et al., 1976; Sørensen et al., 1980; Dooley et al., 1987; Viebrock and Zumft, 1988; SooHoo and Hollocher, 1991; Prudêncio et al., 2000). The isolated *Pseudomonas* sp. was not found in the bands of PCR-DGGE, possibly due to relatively low abundance under acidic conditions (pH 5) (Figure 4C). Anderson and Levine (1986) offered evidence that *Enterobacteriaceae* and *Serratia* sp.'s nitrate respiration produces N_2_O, which was also found in our SC sample. *Enterobacter* sp. was also found as dissimilatory nitrate reduction to ammonium (DNRA) bacteria in agricultural soils (Heo et al., 2020). *Pseudomonas* sp. SC-H2, *Enterobacteriaceae* bacterium SC-L1, and *Serratia* sp. SC-K1 were responsible for N_2_O emissions in our *Sphagnum* samples (SC). These findings suggest that the variation in the N_2_O emission potential of *Sphagnum* found in peatlands is associated with species-specific bacterial communities, which are variable under different species and environments.

### Complex environmental factors also impact N_2_O production of active N_2_O emitters

The top three active N_2_O emitters (*Pseudomonas* sp. SC-H2, *Enterobacteriaceae* bacterium SC-L1, and *Serratia* sp. SC-K1) from SC increased N_2_O production with increasing temperature up to 30°C (Figures 4D–F), illustrating a potential rise in N_2_O emissions following global warming (Pfenning and McMahon, 1997; Voigt et al., 2017a; Chen et al., 2020). For the three active N_2_O emitters, N_2_O production was relatively high at a pH value of 7.0 (Figures 4A–C), which is much higher than the naturally low pH of *Sphagnum* microhabitats (Tahvanainen and Tuomaala, 2003). Although N_2_O reduction to N_2_ by *Pseudomonas* sp. SC-H2 was obvious, the N_2_O production was still high after 5 days of incubation (Figure 6). This result indicated that N_2_O emission hotspots are inclined to be in neutral peatlands, as supported by Palmer and Horn (2015). Combining these results with acetylene inhibition assays at pH value of 5.0 and 7.0 showed that N_2_O reduction to N_2_ was almost negligible at a pH value of 5 for these three active N_2_O emitters. This result is consistent with a previous study of the lack of N_2_O reductase (nos) function at low pH (Liu et al., 2014). This result also suggested that N_2_O reduction was inhibited in the acidic environment in the peat bogs. Since the *Sphagnum* microhabitats are very acidic, N_2_O reductase activity is repressed, supporting that N_2_O reduction is not a pathway decreasing N_2_O emissions in the pristine *Sphagnum* bog system. Under low-pH conditions, N_2_O production by *Pseudomonas* sp. SC-H2 was small, but N_2_O could be accumulated. However, the palsa mounds are formed due to the ice core under the *Sphagnum* peat layer in the subarctic climate, and once they collapse after permafrost thawing, the peat acidity will be neutralized to some extent by mixing with mineral material and minerogenic water flow (Seppälä, 2011; Takatsu et al., 2022).

*Sphagnum* mosses are important for peat accumulation and form a carbon pool of global significance. Increasing atmospheric N deposition can activate phenol oxidase in peat bogs and destabilize peat carbon (Bragazza et al., 2006). Phenol oxidase requires bimolecular oxygen for its activity (Freeman et al., 2004), and drying increases aerobic conditions in peatlands (Swindles et al., 2019) and can degrade recalcitrant phenolic materials. Tahvanainen and Haraguchi (2013) showed that this phenolic mechanism is affected by pH. Such changes may reduce the generally high C:N ratio, which increases net N mineralization, nitrification, and denitrification rates, while subsequently increasing the potential of N_2_O production in peat bogs, while lower C:N ratios ( ≤ 25–30) stimulate N_2_O emissions (Huang et al., 2004; Klemedtsson et al., 2005; Maljanen et al., 2012). Connected mechanisms and the release of ice-trapped N_2_O are further impacted by thawing permafrost (Voigt et al., 2017b). Our findings indicate that N_2_O emissions are not exceptionally high under the naturally cold temperatures and low pH of *Sphagnum* habitats; rather, substantially high pH and temperatures, and perhaps a connected imbalance of microbial communities in such conditions, induced the highest N_2_O emissions. The results warrant caution in interpretation and against unexpected emission potential under rapidly changing conditions. It also calls for a need to monitor the *in situ* N_2_O emissions from different permafrost *Sphagnum species* in the permafrost in future studies.

### Responses of N_2_O emitters to primary metabolites and secondary metabolites of *Sphagnum* mosses

Without sucrose, the N_2_O emitters *Enterobacteriaceae* bacterium SC-L1 and *Pseudomonas* sp. SC-H2 could not emit N_2_O because of their low growth. This result indicated that these two strains were heterotrophic microorganisms that needed to gain C sources from *Sphagnum* moss and form plant-microbial symbionts between plants and microbes. Interestingly, *Serratia* sp. SC-K1 grew well without sucrose and emitted much more N_2_O; meanwhile, it could be significantly inhibited by adding a low concentration of sucrose (0.05%). This result indicated that this strain is an autotrophic microorganism adapted to nutrient-poor environments, using carbon dioxide (CO_2_) as a C source. These autotrophic microorganisms contribute to CO_2_ uptake and carbon sequestration. Drained peatland ecosystems have an immense potential for C sinks to maintain the C balance, even though droughts are occasionally caused by decreasing photosynthesis (Minkkinen et al., 2018).

Our study showed that N_2_O emitters (*Serratia* sp. SC-K1 and *Enterobacteriaceae* bacterium SC-L1) could resist relatively higher concentrations of caffeic acid ( ≤ 0.1 g L^−1^), while the N_2_O emitter (*Pseudomonas* sp. SC-H2) had low resistance to caffeic acid ( ≤ 0.005 g L^−1^) (Figures 5D–F). These results could explain why we could not find the *Pseudomonas* spp. using DGGE band sequencing. Polyphenol (caffeic acid) from *Sphagnum* moss inhibits growth and results in a low relative abundance of *Pseudomonas* spp. The more abundant *Serratia* sp. SC-K1 and *Enterobacteriaceae* bacterium SC-L1 were the dominant N_2_O emitters due to their higher resistance to polyphenolic compounds. The stimulated N_2_O production in the *Sphagnum* moss-microbe vial with 0.1 g L^−1^ caffeic acid confirmed *Serratia* sp. SC-K1 and *Enterobacteriaceae* bacterium SC-L1 were the dominant N_2_O emitters. *Serratia* spp. are gram-negative bacilli and belong to the family *Enterobacteriaceae*. The interaction of polyphenolic compounds and *Enterobacteriaceae* bacteria might directly influence N_2_O emissions in peatland ecosystems. High concentrations of polyphenols are likely to lower N_2_O emissions. The response of phenol oxidase to N deposition differs by ecosystem type. In peat bogs, elevated N deposition decreased polyphenols' contents and decreased the polyphenol ratio to N, which may increase N_2_O production due to an inverse relationship between N_2_O emissions and the polyphenol to nitrogen ratio (Pimentel et al., 2015).

### N_2_O production of active N_2_O emitters

The three N_2_O emitters preferred KNO_3_ as a substrate over NH_4_Cl. This result suggested that these three isolates mainly use DNRA or denitrification to produce N_2_O gas. For the *Enterobacteriaceae* bacterium SC-L1 and *Serratia* sp. SC-K1, the *nirS, nirK*, and *nosZ* genes were not detected, but the *narG* gene was, suggesting that they do not have nitrite reductase and are non-denitrifiers consistent with other *Enterobacteriaceae* bacteria emitting N_2_O as a final product (Arkenberg et al., 2011). *Enterobacter* species are often reported as producing N_2_O by DNRA (Smith and Zimmerman, 1981). This result indicated that they are also important sources for N_2_O emissions in SC dominant bogs. *Pseudomonas* sp. SC-H2 harbored *nosZ, nirS*, and *narG*. Therefore, *Pseudomonas* sp. SC-H2 was a typical denitrifier. Microbial heterotrophic denitrification and DNRA compete for shared resources (Jia et al., 2020).

Although the N_2_O potential was relatively high in the SC sample, the N_2_O emissions *in situ* in the peat bogs were generally low in northern Finland, which might be impacted by the complexity of environmental conditions (Dinsmore et al., 2017). The potential N_2_O emissions in the field (Repo et al., 2009; Voigt et al., 2017b) and laboratory incubations (Elberling et al., 2010) increase with increasing mineral N availability, permafrost thawing, and drainage. A previous study suggested that drainage of bogs alters nutrient cycling and microbial communities to increase N_2_O emissions (Frolking et al., 2011). Unvegetated (free of vascular plants) peat surfaces resulting from wind erosion and frost action were hot spots for N_2_O emission in subarctic permafrost peatlands due to the absence of plant nitrogen uptake, a low C:N ratio, and sufficient drainage (Marushchak et al., 2011; Voigt et al., 2017b). *Pseudomonas* sp. SC-H2 had negligible N_2_O emissions at low pH (<4.5), while the other two N_2_O-emitting *Enterobacteriaceae* bacteria from SC exhibited contrasting patterns in the *Sphagnum* bogs. Therefore, the contribution of denitrification and DNRA to N_2_O emissions in boreal peat bogs should be considered in future studies.

## Conclusion

In summary, our study identified several N_2_O emitters in microbial communities of *Sphagnum* samples from the subarctic permafrost habitat of palsa mires. A composite sample of SC showed high potential to emit N_2_O, and a composite of SF showed moderate potential to emit N_2_O. The N_2_O emission potential was attributed to distinctive bacterial communities inhabiting moss leaves in both cases. Two classes of hyperactive N_2_O emitters hidden in the SC holobiont were revealed. *Pseudomonas* sp. SC-H2 was found to harbor *narG, nirS*, and *nosZ* genes. N_2_O reduction to N_2_ catalyzed by N_2_O reductase was noteworthy in the neutral pH microenvironment. The other hyperactive N_2_O emitters, *Enterobacteriaceae* bacterium SC-L1 and *Serratia* sp. SC-K1 lacked the *nirS, nirK*, and *nosZ* genes but contained the *narG* gene and emitted NO/N_2_O as the final product, possibly *via* the DNRA pathway. These findings provided some theoretical evidence for the future N_2_O emission study of the *in situ* subarctic palsa under elevated N availability and global warming.

## Data availability statement

The datasets presented in this study can be found in online repositories. The names of the repository/repositories and accession number(s) can be found in the article/Supplementary material.

## Author contributions

YH and YN designed the research, experiments, and acquired the funds. YH, RI, and TT collected the samples in Finland. YN performed experiments and analyzed data. YN, SYL, XT, XL, SL, TT, RI, and QY wrote and edited the paper. All authors read and approved the final manuscript.

## Funding

This research was supported by the National Natural Science Foundation of China (32071596 to YN), the Key Special Project for Introduced Talents Team of Southern Marine Science and Engineering Guangdong Laboratory (Guangzhou) (GML2019ZD0408), Grants-in-Aid A (20255002 and 26252058 to YH) and B (26304042 to YH) by JSPS (Japan Society for the Promotion of Science). Kilpisjärvi Biological Station of the University of Helsinki supported our fieldwork. We sincerely appreciate the Chinese Scholarship Council for a scholarship to YN (CSC 201204910200).

## Conflict of interest

The authors declare that the research was conducted in the absence of any commercial or financial relationships that could be construed as a potential conflict of interest.

## Publisher's note

All claims expressed in this article are solely those of the authors and do not necessarily represent those of their affiliated organizations, or those of the publisher, the editors and the reviewers. Any product that may be evaluated in this article, or claim that may be made by its manufacturer, is not guaranteed or endorsed by the publisher.
