# Supplementary material for: Sphagnum capillifolium holobiont from a subarctic palsa bog aggravates the potential of nitrous oxide emissions
Source: Front Plant Sci. 2022 Sep 7;13:974251. doi: 10.3389/fpls.2022.974251 (PMC9490422; doi:10.3389/fpls.2022.974251)
Supplement: Supplementary file 1 [file Data_Sheet_1.docx]

***Sphagnum capillifolium* holobiont from a subarctic palsa bog aggravates the potential of nitrous oxide emissions**

**Yanxia Nie^1,2,5,^*,** **Sharon Yu Ling** **Lau^3,5^,** **Xiangping Tan^1^, Xiankai Lu^1^, Suping Liu^1^,** **Teemu** **Tahvanainen^4^,** **Reika Isoda^5^,** **Qing** **Ye^1,2^, Yasuyuki** **Hashidoko^5^**^※^

^1^Key Laboratory of Vegetation Restoration and Management of Degraded Ecosystems, South China Botanical Garden, Chinese Academy of Sciences, Guangzhou 510650, China.

^2^Southern Marine Science and Engineering Guangdong Laboratory, Guangzhou 511458, China.

^3^Sarawak Tropical Peat Research Institute, Kuching-Samarahan Expressway, Kota Samarahan, 94300, Malaysia.

^4^Department of Environmental and Biological Sciences, University of Eastern Finland, Joensuu FI-80100, Finland.

^5^Graduate School of Agriculture, Hokkaido University, Sapporo 060-0808, Japan.

^※^Deceased

*** Correspondence:**

Corresponding Author: Dr. Yanxia Nie

Email: [nieyanx@scbg.ac.cn](mailto:nieyanx@scbg.ac.cn)

South China Botanical Garden, Chinese Academy of Sciences, Xingke Road 723, Tianhe District, Guangzhou, 510650, China.


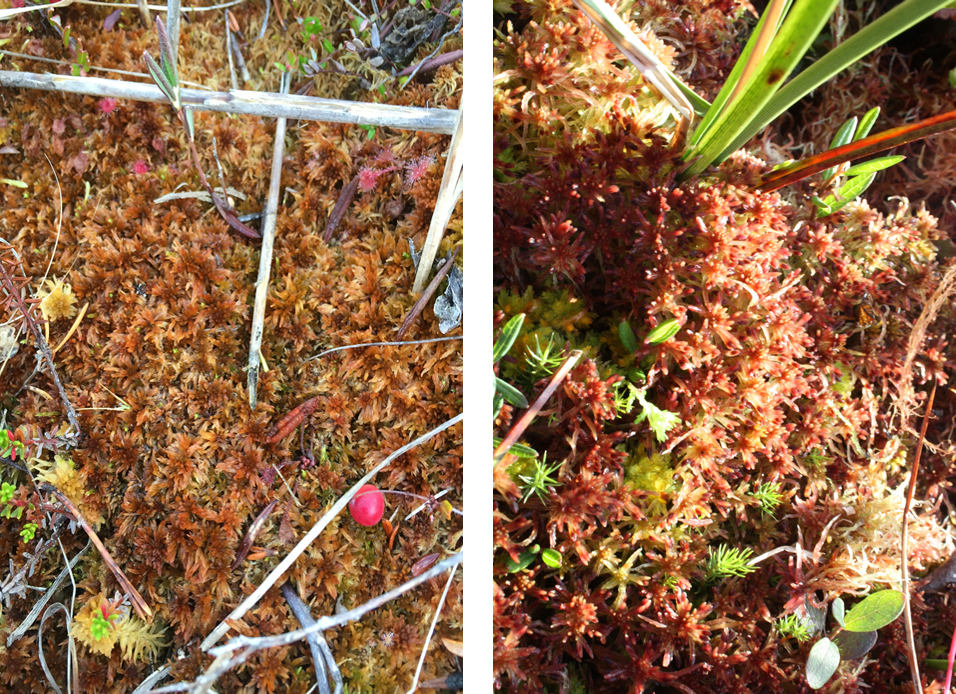


*Sphagnum fuscum*   *Sphagnum capillifolium*

Fig. S1 Vegetation of *Sphagnum* mosses in a plateau of a permafrost mound of a palsa mire near Kilpisjärvi (68° 52’ 45; 21° 4’ 40), Finland. Two characteristic species including *Sphagnum fuscum* and *Sphagnum capillifolium* were collected.


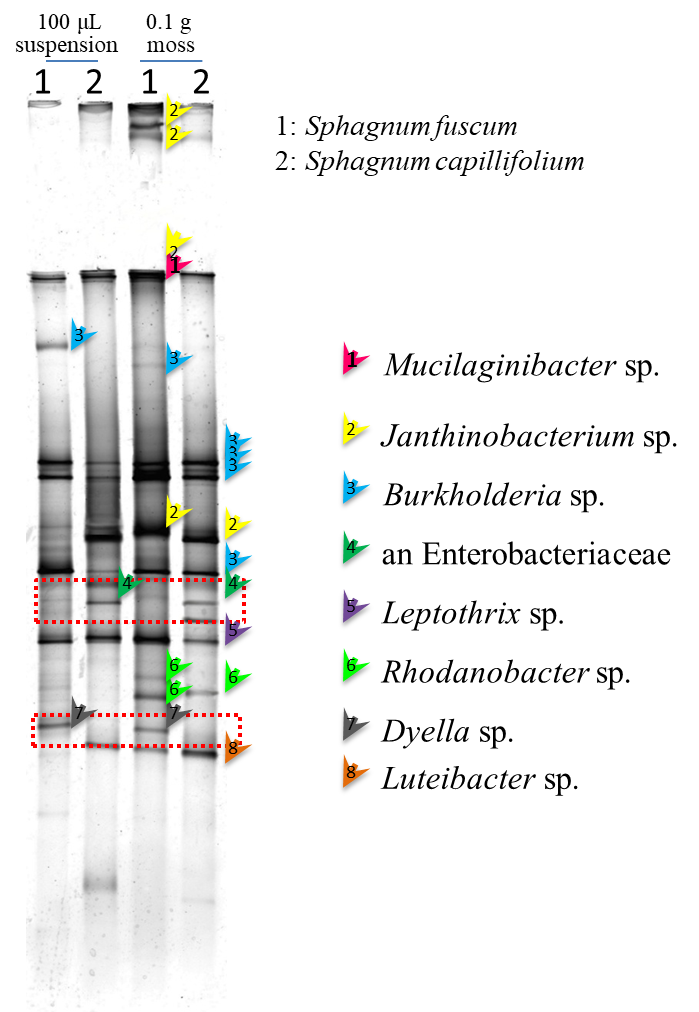


Fig. S2. The profile of DGGE of the two *Sphagnum* species


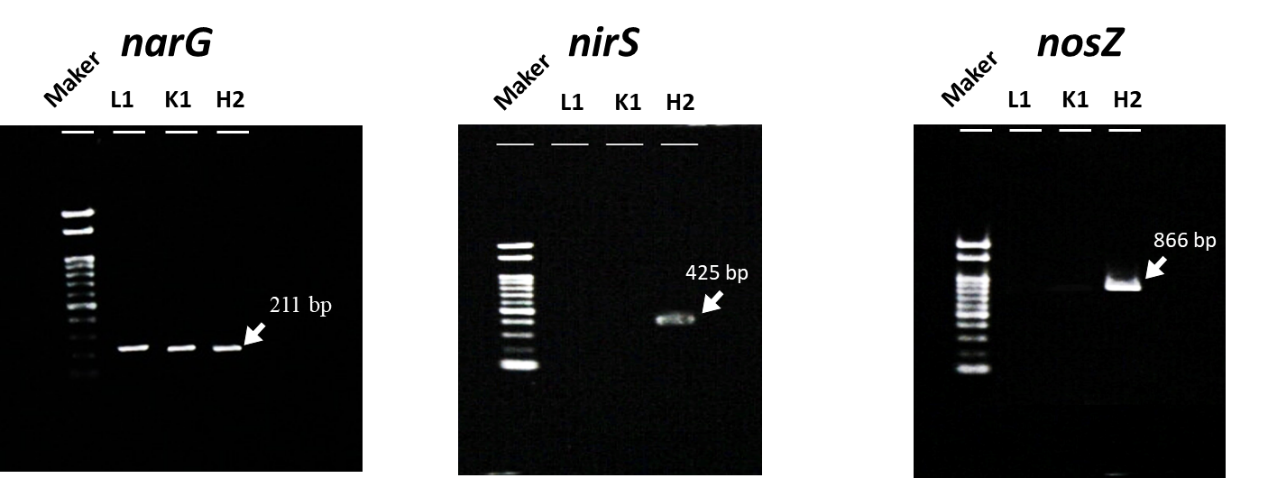


Fig. S3. Detection of *nar*G, *nir*S, and *nos*Z genes of the three active N_2_O emitters.


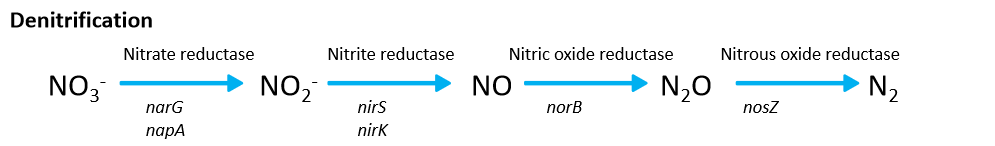


Fig. S4. Functional genes in the process of denitrification.

| **Target**  **gene** | **Primer set** | **Sequence (5'–3')** | **Thermal profile** | **Reference** |
| --- | --- | --- | --- | --- |
| *nar*G | 2168F  2391R | 5‘-TCG GGC AAG GGC CAC GAA TAC-3‘  5‘-TTC TCG TAC CAC GTC GCG GTC-3‘ | 95˚C 10 min, 30 cycles of 52˚C 1 min, 72˚C 1 min, 72˚C 10 min | This study |
| *nir*S | cd3AF  R3cd | 5‘-GTSAACGTSAAGGARACSGG-3‘  5‘-GASTTCGGRTGSGTCTTGA-3‘ | 95˚C 10 min, 30 cycles of 52˚C 1 min, 72˚C 1 min, 72˚C 10 min | Throbäck et al., 2004 |
| *nos*Z | 661F  1527R | 5‘-CGG CTG GGG GCT GAC CAA-3‘  5‘-CTG RCT GTC GAD GAA CAG-3‘ | 95˚C 10 min, 30 cycles of 55˚C 1 min, 72˚C 1 min, 72˚C 10 min | Scala and Kerkhof, 1998 |

Table S1 The detail reaction conditions of PCR amplifications of *nar*G, *nir*S, *nos*Z genes.

Table S2 Screening of the N_2_O emitters isolated from the surface of *Sphagnum* mosses leaves. Incubation conditions of these isolated pure strains: pH=5, incubated at 15˚C, 5 days, n=3, with 0.05% sucrose. (-) indicated inactive N_2_O emitters, (+) indicated active emitters.

| *Sphagnum* species | Bacterium | Most aligned sequences | N_2_O (ng vial^-1^ d^-1^) | N_2_O emitters |
| --- | --- | --- | --- | --- |
| SF | SF-B1 | *Burkholderia* sp. | 62.12 | - |
|  | SF-D2 | *Burkholderia* sp. | 54.02 | - |
| SC | SC-L1 | *Enterobacteriaceae.* | 338.49 | + |
|  | SC-K1 | *Serratia* sp. | 186.22 | + |
|  | SC-H2 | *Pseudomonas* sp. | 427.35 | + |
|  | SC-M1 | *Burkholderia* sp. | 51.90 | - |
